# Using Rapid Chlorophyll Fluorescence Transients to Classify *Vitis* Genotypes

**DOI:** 10.3390/plants9020174

**Published:** 2020-02-01

**Authors:** Jorge Marques da Silva, Andreia Figueiredo, Jorge Cunha, José Eduardo Eiras-Dias, Sara Silva, Leonardo Vanneschi, Pedro Mariano

**Affiliations:** 1 Biosystems and Integrative Sciences Institute (BioISI), Faculdade de Ciências, Universidade de Lisboa, 1749-016 Lisboa, Portugal; aafigueiredo@fc.ul.pt (A.F.); plmariano@fc.ul.pt (P.M.); 2 National Station of Viticulture and Enology, 2565-191 Dois Portos, Portugal; jorge.cunha@iniav.pt (J.C.); eiras.dias@iniav.pt (J.E.E.-D.); 3 LASIGE, Faculdade de Ciências, Universidade de Lisboa, 1749-016 Lisboa, Portugal; sgsilva@fc.ul.pt (S.S.); lvanneschi@fc.ul.pt (L.V.); 4 NOVA Information Management School (NOVA IMS), Universidade Nova de Lisboa, Campus de Campolide, 1070-312 Lisboa, Portugal; lvanneschi@novaims.unl.pt (L.V.)

**Keywords:** Kautsky effect, k-nearest neighbors, decision trees, artificial neural networks, genetic programming, molecular markers, *Vitis*, chlorophyll a fluorescence, photosynthesis

## Abstract

When a dark-adapted leaf is illuminated with saturating light, a fast polyphasic rise of fluorescence emission (Kautsky effect) is observed. The shape of the curve is dependent on the molecular organization of the photochemical apparatus, which in turn is a function of the interaction between genotype and environment. In this paper, we evaluate the potential of rapid fluorescence transients, aided by machine learning techniques, to classify plant genotypes. We present results of the application of several machine learning algorithms (k-nearest neighbors, decision trees, artificial neural networks, genetic programming) to rapid induction curves recorded in different species and cultivars of vine grown in the same environmental conditions. The phylogenetic relations between the selected *Vitis* species and *Vitis vinifera* cultivars were established with molecular markers. Both neural networks (71.8%) and genetic programming (75.3%) presented much higher global classification success rates than k-nearest neighbors (58.5%) or decision trees (51.6%), genetic programming performing slightly better than neural networks. However, compared with a random classifier (success rate = 14%), even the less successful algorithms were good at the task of classifying. The use of rapid fluorescence transients, handled by genetic programming, for rapid preliminary classification of *Vitis* genotypes is foreseen as feasible.

## 1. Introduction

The chlorophyll fluorescence emitted in vivo by photosynthetic systems subjected to a rapid dark–light transition follows a typical pattern, named the Kautsky effect. In fact, this phenomenon was first described by Kautsky and Hirsch [1], who speculatively correlated it with the onset of carbon metabolism. However, the recording of continuous fluorescence only became a key tool for photosynthesis research when fluorometers were furnished with high time resolution capabilities, allowing researchers to explore the kinetics of the fast fluorescence rise in dark–light transitions, and to underline its polyphasic nature. The commercial release of the Plant Efficiency Analyzer by the UK-based maker Hansatech made this technique broadly available to plant physiologists and breeders [2]. In the meantime, the theoretic basis for the analysis of these signals was given, and the JIP-test (conceived to assess the efficiency of plants’ photochemical apparatus, and termed after the main inflections in the fast fluorescence rise, O, J, I, and P) was launched [3].

Several indexes and parameters representing the energy flow in PS II (photosystem II) photochemical reactions can be computed based on the measurement of the fast transient of Chl a fluorescence [4,5]. The OJIP transient reflects the sequential but overlapping reduction of the electron acceptor pool of PS II [6] and can be used to acquire information on the stoichiometry of the elements of the photosynthetic electron transport chain, their redox state, and the relative PS II antenna size [7], which result from the interaction between plants’ genotype and the environment (G x E). The OJIP transient proved to be very responsive to stress caused by different environmental factors [8,9,10,11,12,13]. Thus, this technique has been extensively used in low throughput plant phenotyping (Costa et al. [14] and references therein) to phenotype the physiological status of plants, i.e., to position the phenotype along the “condition or situation axis” of the conceptual phenotype space proposed by the USDA/NSF [15]. However, Tyystjarvi et al. [16] suggested that the Kautsky curve is a built-in bar code that can be used in the automatic identification of plant species, when coupled with artificial intelligence techniques. This means that, if plants occupy the same position on the “condition or situation axis” of the phenotype space, Kautsky curves may be used to position a specimen on its “taxon or genotype axis”. Tyystjarvi et al. [16] reported high success rates in identifying different species and clustering them according to their phylogenetic origin, using different machine learning algorithms. However, no further references were found in the literature. The recent interest in plant phenotyping, nonetheless, brought new attention to this subject. 

Grapevine (*Vitis vinifera* L.) is one of the most widely cultivated and economically important fruit crops in the world, reaching 7.6 mha of planted vineyards in 2016 and a global wine production of 250 mhL in 2017. Portugal is one of the top producers and exporters, with over 194 kha of cultivated area [17]. In Portugal, 343 grapevine cultivars are legally accepted for wine production [18]; 236 of them are considered autochthonous and 107 belonging to foreign germplasm. This legal status resulted from a thorough characterization and analysis of the National Ampelographic Collection (NAC), including the use of morphological descriptors and molecular markers such as nuclear microsatellite and single-nucleotide polymorphisms [19,20,21,22,23]. Certification of grapevine varieties is crucial for producers, and conventional ampelometric methods that require visual inspection and the measurement of precise phenotypic features of grapevines—mainly the leaf characteristics, or DNA based methods [24]—are laborious and very time consuming.

In this work, we aimed to investigate the suitability of Kautsky curves, aided by machine learning techniques, in distinguishing between different closely related species and different cultivars of the same species, testing the possibility of adapting the JIP test to high throughput grapevine phenotyping. The success of automatic genotype classification will be presented and discussed in face of the phylogenetic distance between genotypes. The feasibility of using rapid fluorescence transients connected to machine learning algorithms in high throughput plant platforms will be evaluated.

## 2. Results

### 2.1. Genetic Analysis

**Figure 1:**
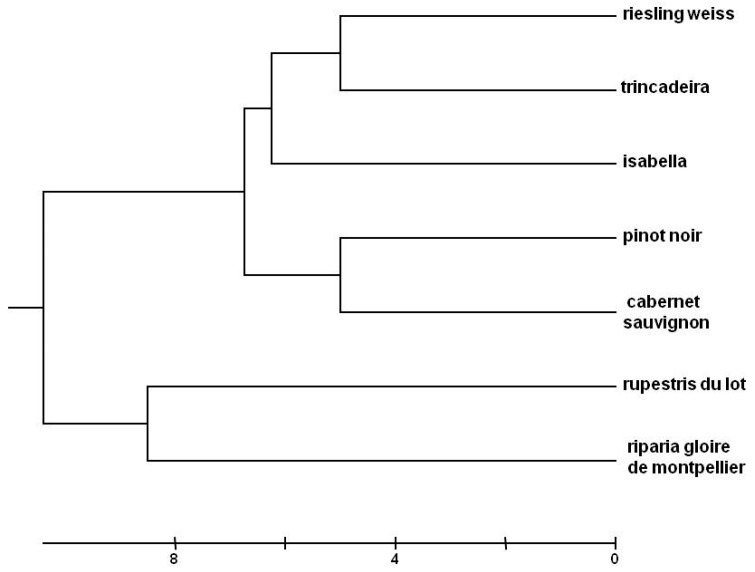
Phenogram of the genotyped *Vitis* samples based on the unweighted pair group method with arithmetic mean averages with a squared distances matrix, generated with allelic data from the nine SSR polymorphisms analyzed.

Nuclear simple sequence repeats (SSRs) or nuclear microsatellites are widely used to access the genetic diversity of grapevines [25,26] and to confirm the genotype identity. The set of nine nuclear SSR Markers used (VVS2, VVMD5, VVMD7, VVMD25, VVMD27, VVMD28, VVMD32, VRZAG62, VRZAG79) confirm the identity of the grapevine accessions used in this study and infer their genetic dissimilarities. In the phenogram, there is a clear separation between the different grapevine species (Figure 1). Statistical analysis grouped the interspecific hybrid Isabella with *Vitis vinifera* cultivars.

### 2.2. Machine Learning

A random classifier has a 14% chance of correctly classifying a sample. This is the baseline used for comparing the success rates of the classifiers obtained with the four methods selected.

Table 1 displays the success rates and parameterization of the four methods used. K-nearest neighbors (KNN) achieved an overall accuracy of 58.5%, clearly revealing that there is structure in the features that allows for the induction of a predictive model, but decision trees (DT) achieved only 51.6% overall accuracy with their simple models, which called for more powerful methods. Neural networks (NN) and genetic programming (GP) reached overall accuracies of 71.8% and 75.3%, respectively.

**Figure 2:**
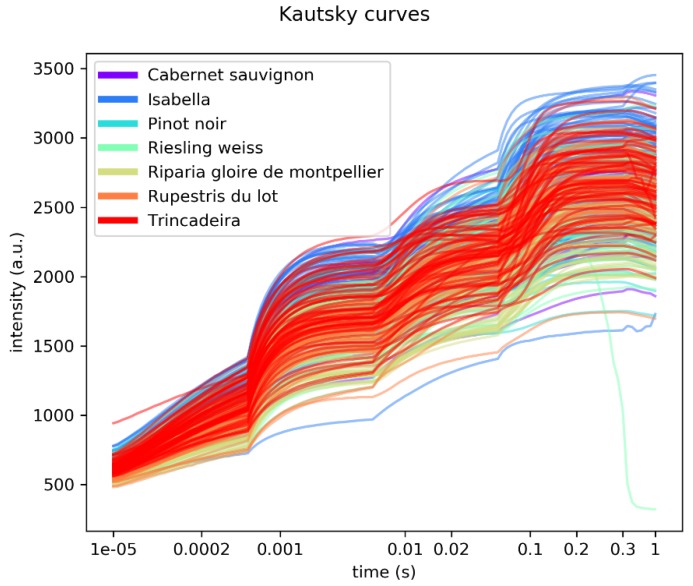
Overlap of all rapid fluorescence induction curves measured.

Figure 2 shows the superimposition of all rapid fluorescence induction curves recorded.

The curves show the four typical phases: O, J, I and P. There is a substantialoverlapping of the different genotypes, although Isabella and Riparia Gloire de Montpellier show a tendency towards higher and lower values, respectively.

**Figure 3:**
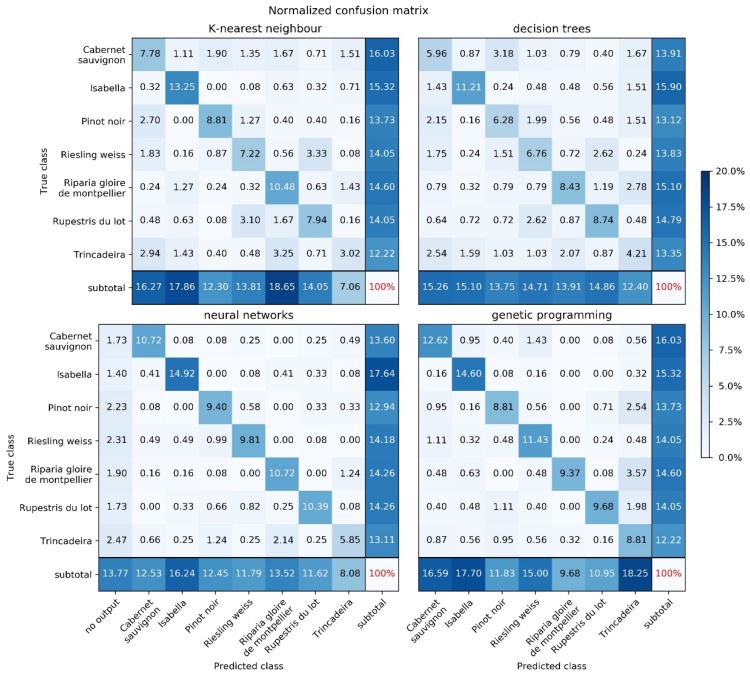
Confusion matrices for all the methods, using the parameterizations specified in Table 1.

Figure 3 shows the normalized confusion matrices obtained by the four methods on the test data. The percentages shown are calculated by (1) obtaining the results for each of the 30 random test sets, (2) aggregating the results by summing the 30 numbers corresponding to each matrix cell, and (3) dividing each cell by the sum of all the numbers in the aggregate matrix, i.e., by the total number of predictions made for all 30 test sets. Since the test sets are obtained by uniform random sampling, the distribution of classes within each test set is not the same for different methods (except for KNN and GP, which used the exact same data partitions). All the methods revealed the cultivar that is more easily classified (Isabella), and most (except GP) agreed on the cultivar that is more difficult (Trincadeira).

**Figure 4:**
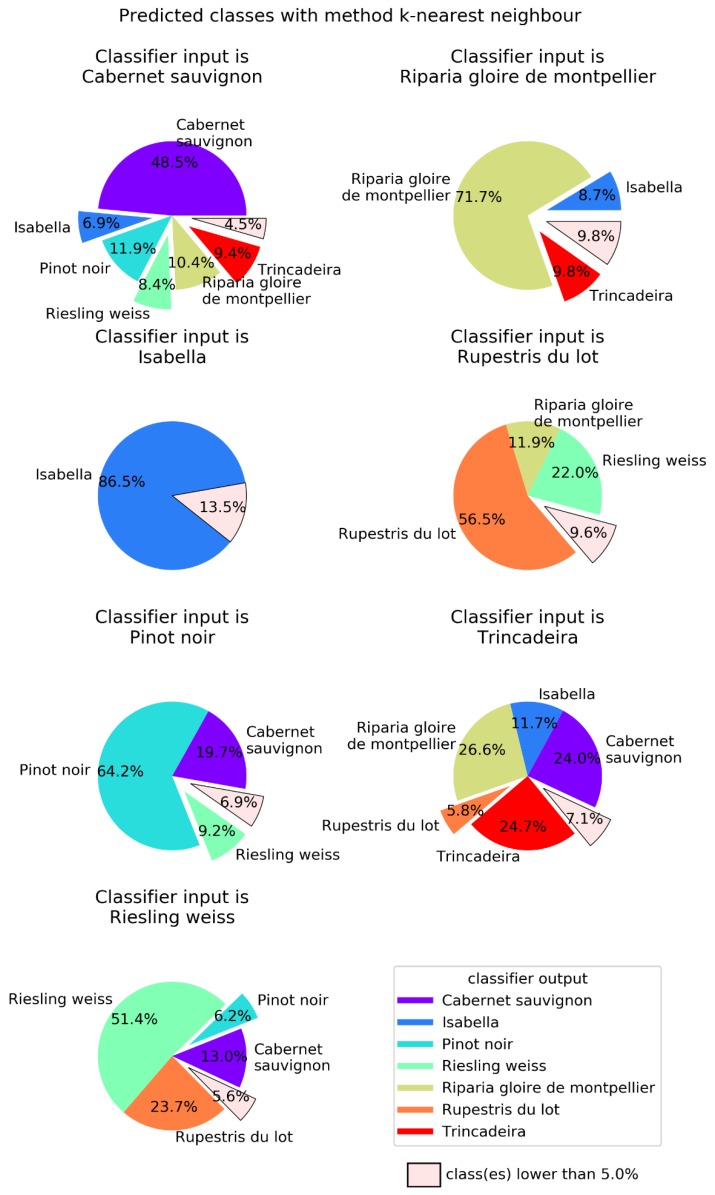
K-nearest neighbors (KNN) output for each of the possible seven input *Vitis* genotypes. KNN used k = 5 as the number of neighbors with which to perform the classification. Global success rate is 58.5%.

**Figure 5:**
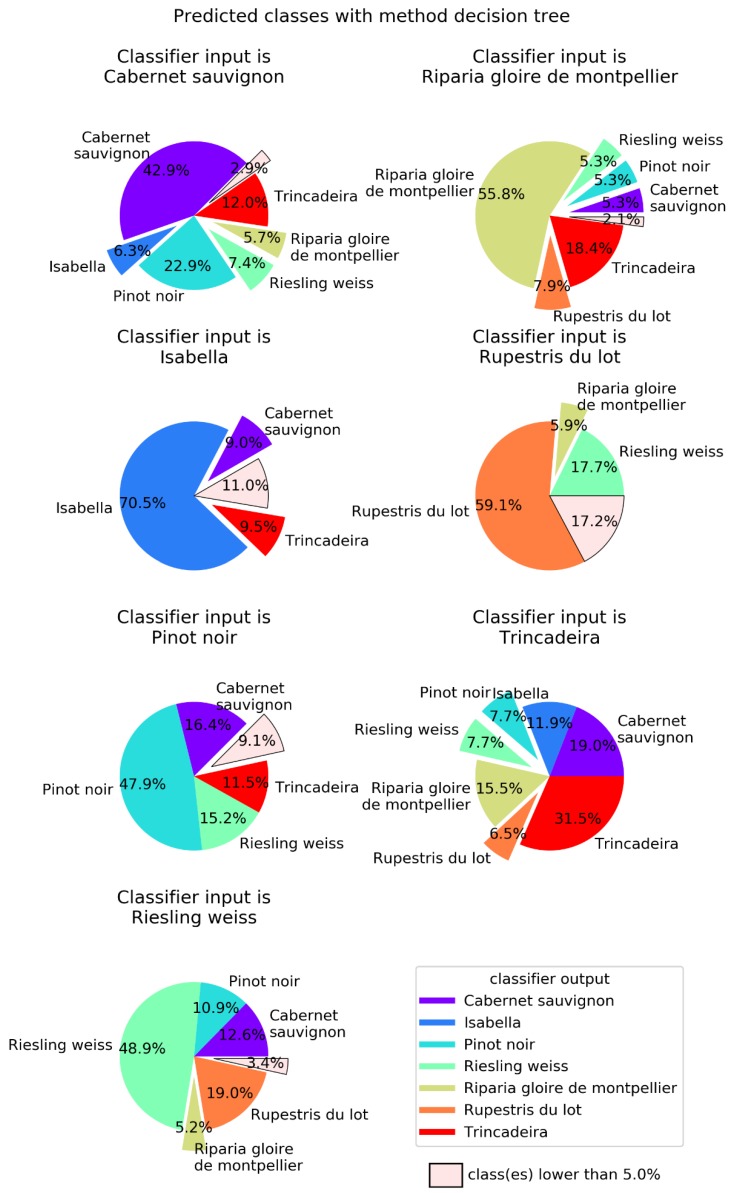
Decision trees (DT) output for each of the possible seven input *Vitis* genotypes. DT were defined using the entropy criterion, a maximum depth of 19 and a minimum number of samples of five. Global success rate is 51.6%.

**Figure 6:**
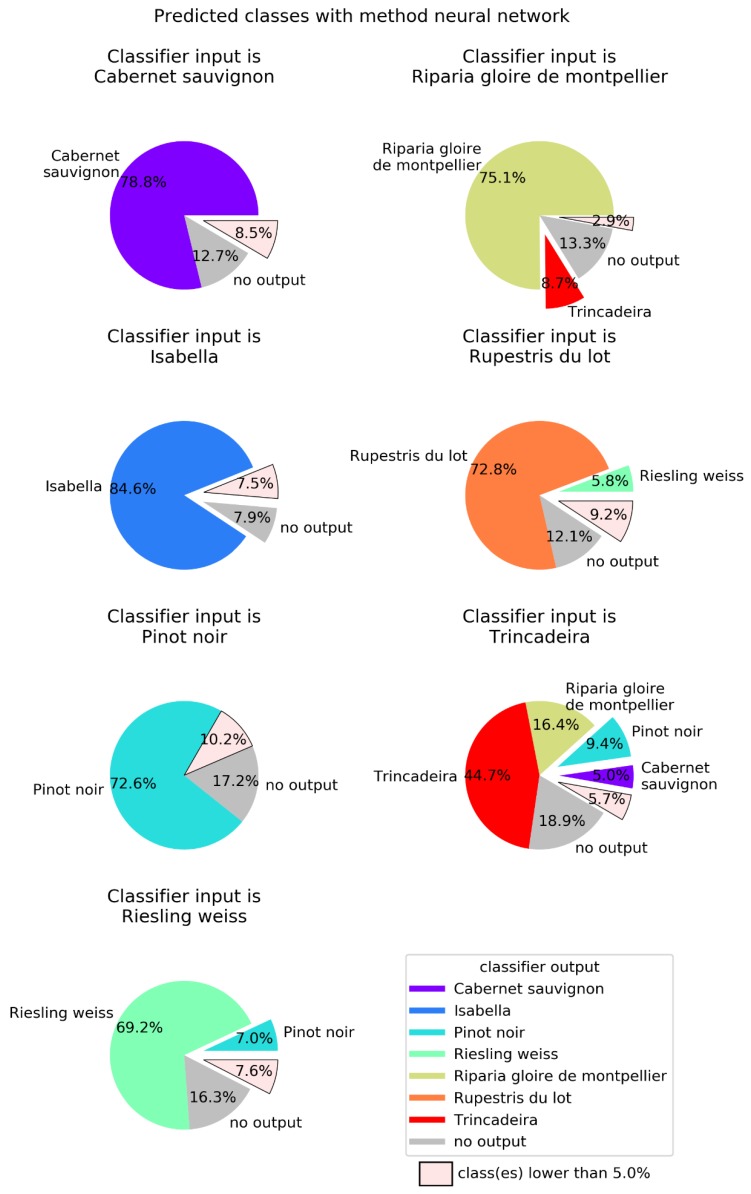
Neural networks (NN) output for each of the seven possible inputs. NN were defined with 5000 neurons, a single hidden layer, and the neurons in the hidden layer used the logistic activation function. Global success rate is 71.8%.

**Figure 7:**
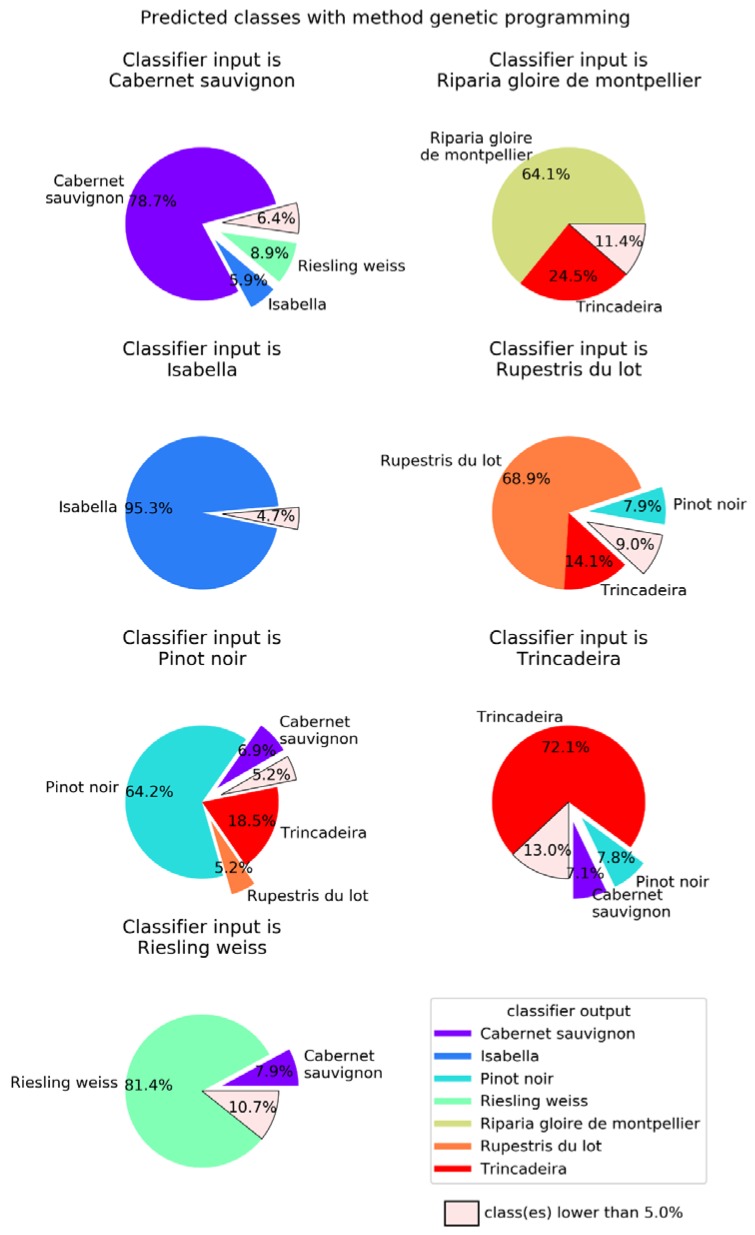
Genetic programming (GP) classifier output for each of the seven possible inputs. Results were obtained using a population of 250 individuals allowed to evolve for at most 100 generations. Global success rate is 75.3%.

In order to better visualize the confusion between cultivars, Figure 4, Figure 5, Figure 6 and Figure 7 show pie charts of the predicted cultivars for each expected (real) cultivar, for the methods KNN, DT, NN, and GP, respectively. Focusing on the two best methods, NN (Figure 6) determined the class with the best success rate is Isabella with 84.6%. Regarding the cultivars with a lower success rate, we have Trincadeira with 44.7%. We can also see pairs of classes that are often confused between each other: (1) Trincadeira and Riparia Gloire de Montpellier; (2) Pinot Noir and Riesling Weiss; (3) Pinot Noir and Rupestris du Lot. There is a particularity in the predictions of the NN we used, which results in “no output” when the output signal of the network is not strong enough. 

When compared to the multilayer perceptron, GP has a higher global success rate (75.3%), but not in all classes (Figure 7). The class with best success rate is Isabella with 95.3%. Regarding the cultivars with lower success rates, we have Riparia Gloire de Montpellier and Pinot Noir, with 64.1% and 64.2%, respectively. Trincadeira, which had been classified by NN with 44.7% accuracy, achieved 72.1% with GP. Trincadeira is the cultivar with the highest misclassification rate, e.g., with Riparia Gloire de Montpellier, Pinot Noir and Rupestris du Lot.

**Figure 8:**
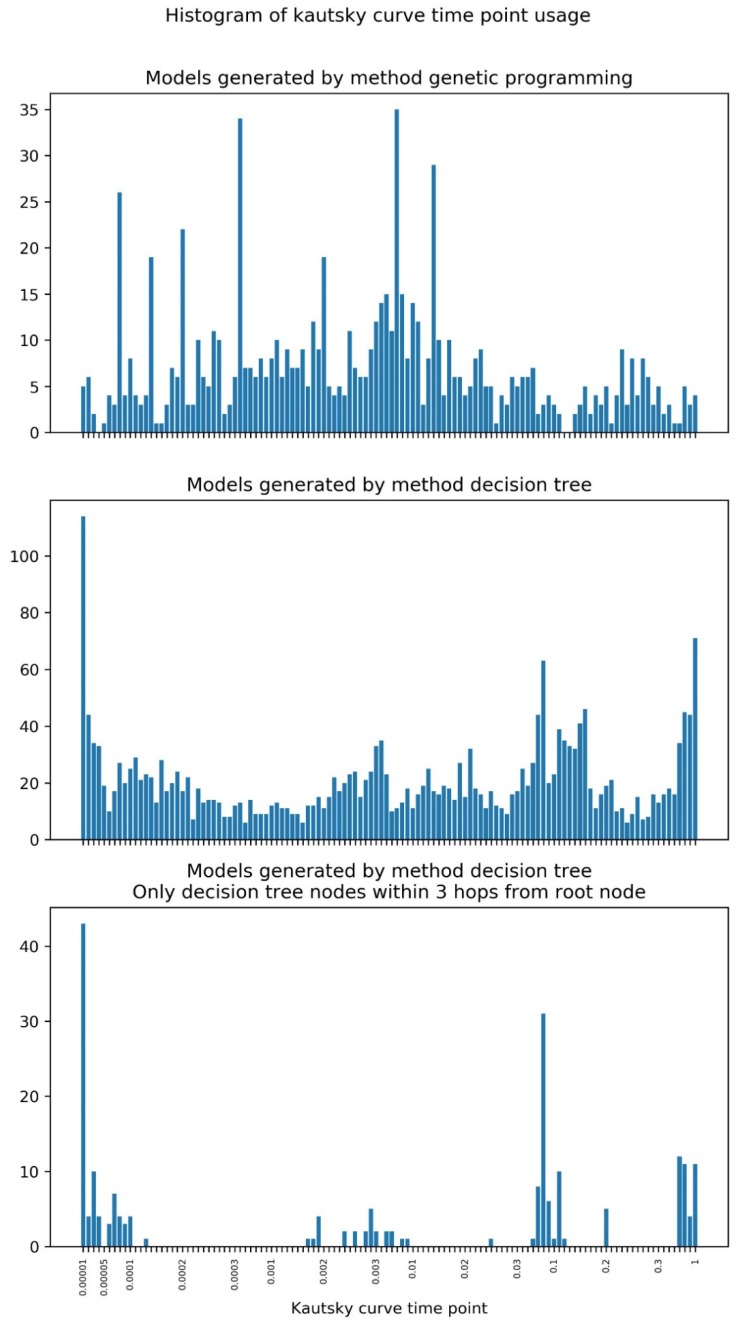
Histogram of Kautsky curve time point usage in the DT and GP models obtained.

Figure 8 shows the histogram of Kautsky curve time points usage in GP and DT models. 

In the case of GP, some points are used much more often than the others, corresponding to 0.08, 0.14, 0.2, 0.4, 2, 7 and 14 ms. The points more frequently used by DT are packed in four groups: 0.01 to 0.13, 1.7 to 9, 25 to 200, and 700 to 1000 ms.

## 3. Discussion

All four learning methods presented good capabilities for automatically recognizing a certain *Vitis* phenotype against the background of the seven phenotypes/genotypes studied. However, intergenotypic variation was significant, with success rate varying among 24.7%, 31.5%, 44.7% and 72.1% for Trincadeira, and among 86.7%, 70.5%, 84.6% and 95.3% for Isabella, when KNN, DT, NN and GP were used, respectively. Trincadeira was mostly misclassified as Cabernet Sauvignon (24% and 19%) when KNN and DT were used, respectively, and it was mostly misclassified as Riparia Gloire de Montpellier (26.6% and 16.4%) by KNN and NN, respectively, and as Pinot Noir (7.8%) by GP. Although GP had no major problems in correctly classifying Trincadeira, when classifying Riparia Gloire de Montpellier it wrongly predicted Trincadeira in 24.5% of the cases. However, the genetically closest cultivar to Trincadeira is Riesling Weiss (Figure 1) (misclassification rate = 3.9% with KNN, 7.7% with DT, 1.9% with NN and 4.5% with GP), and then Isabella (misclassification rate = 11.7% with KNN, 11.9% with DT, 1.9% with NN and 4.5% with GP). Therefore, the success rate of the classification is not primarily determined by the genetic distance of the genotypes, contrary to the findings of Tyystjarvi et al. [16]. Several factors may explain this difference. On one hand, Tyystjarvi and collaborators [16] compared different species that were always from a different genus, some of them also belonging to different higher taxa. In our case, we compared species within the same genus and cultivars within the same species, i.e., we used a set of much closer genotypes. Significant genetic distances may influence classification success [27], but this effect might be lost among smaller genetic differences, where confounding effects may prevail. In fact, although all our measurements were made in the same experimental field, micro-environmental differences may exist and contribute to the production of different functional phenotypes. Furthermore, the genetic proximity of the species and cultivars was calculated using nuclear molecular markers, not related to photosynthetic apparatus. Besides, although some of the macromolecular photosynthetic subunits are encoded by nuclear genes, synthesized on cytoplasmic ribosomes and imported into the chloroplast where they are assembled, others are encoded by chloroplast genes and translated on chloroplast ribosomes, being assembled, with their nuclear-encoded partners, into functional complexes [28,29]. Therefore, chloroplast genome phylogeny may not entirely overlap with nuclear markers phylogeny. Nonetheless, conclusions are partly limited by the low number (7) of genotypes used. Even though these were selected in order to reflect the diversity of *Vitis* germplasm (three species, one interspecific hybrid and four cultivars within one species), the number of cultivars should be increased in future studies, taking advantage of the richness of *Vitis* germplasm [18]. Also, in our experiment, models were trained and tested on leaf samples from the same experimental field, minimizing the effect of environmental influence. Expectedly, the trained models would have worse performance for data collected in other locations and years, due to confounding environmental factors.

As seen above, both NN and GP performed much better than KNN and DT. While NN require parameter fine-tuning in order to find a model with good success rate, DT are not affected by this problem [30], and neither is GP [31,32]. However, DT have a lower success rate, which is a disadvantage in a production system. Parameter fine tuning in NN is a time-consuming process: training the NN with 5000 neurons took one day compared to 15 min for obtaining all the DT presented in this paper, and even less for KNN. Nevertheless, with an overall success rate of 71.8%, it is worth the time spent in NN training. An advantage is the fact that most of the unsuccessful classifications result in “no output”, meaning that erroneous classifications are avoided. The GP method provided the best success rate, 75.3%, mostly due to improvement in the classification of Trincadeira. Although not a standard procedure, GP could also have been coached to predict “no output” when the prediction is not very reliable, which could further improve its success rate. While GP is not a fast method in general, in this problem we obtained all the classifiers within 3 hours. This happened because this classification task was perceived by the algorithm to be an easy one, causing the evolved models to remain very simple along the entire evolution. Another advantage of GP is that its models, like the ones of DT, are perfectly readable (white-box models), whereas the ones obtained by NN are almost impenetrable (black-box models).

The white-box models can provide interesting insight into which points of the Kautsky curve are being used in the classification task (Figure 8). In the case of GP, some points are used much more often than others, corresponding to 0.08, 0.14, 0.2, 0.4, 2, 7 and 14 ms. In general, the range corresponding to 3 to 11 ms has a higher density of used points. All these points belong to the initial phases of the Kautsky curve O and J, which are related to energy absorption by the photosynthetic antenna (O) and energy migration within the antenna to the PS II reaction center (RC) (J). The points that are used most often by both models are different depending on whether we used all DT nodes or just the ones closest to the root node. Considering only the latter (nodes farther away are a sign of specialization), we observe that the points more frequently used by DT are packed in four groups: 0.01 to 0.13, 1.7 to 9, 25 to 200, and 700 to 1000 ms. The first two groups correspond to the phases O and J of the Kautsky curve and are shared with GP. The third and fourth group include points corresponding to the phases I (related with charge separation at the RC) and P (related with the onset of photosynthetic electron transport), showing that DT makes use of information from the entire fluorescence induction process, in contrast with GP. Most indices calculated from Kautsky curves (e.g., PI, the Performance Index) are computed with fluorescence emission at only four time points: 0.05 (taken as the minimal fluorescence emission, Fo), 0.3 and 2 ms, and the fluorescence at point P, which may be reached at different times. The first three points are included in the range of values used by both GP and DT, whereas the latter is used only by DT.

The use of OJIP curves in high throughput plant phenotyping (HTPP) poses some a priori problems. Although some information may be obtained from the application of the JIP-test to pre-illuminated leaves, the most used protocols require a dark-adaptation period [33], which is the main hindrance to the use of the JIP-test on high throughput automated plant phenotyping. On the other hand, individual measurements are rapid, as usually a 1 s light (saturating) pulse is applied, and the kinetics of fluorescence rise is immediately recorded. Although this technique has proved useful in manual low throughput plant phenotyping [13,34] none of the commercial phenotyping platforms make use of it. It is possible to envisage, however, a system where whole plants would be pre-adapted for dark conditions, and non-contact measurements of the fluorescence induction curve would be made. In fact, imaging of the JIP parameters is already possible and has been used to screen wild barley genotypes under heat stress [35]. Even though, in most situations, the aim of high throughput plant phenotyping is to characterize the impact of a stressor (e.g., heat) in the plant’s phenotype (across a range of genotypes), in plant breeding it is frequently useful to phenotypically discriminate genotypes. This is the case, for instance, in the grouping of the F1 generation according to the similarity of each one of the progenitors. Our group have evidence that other optical non-invasive techniques (e.g., laser induced fluorescence and reflectance spectroscopy) are more suited to the stochastic automatic identification of plant genotypes and plant physiological conditions, including in *Vitis* [36]. However, the JIP curve pertains more valuable information about the function of plants’ photochemical apparatus and might prove useful as a complementary diagnostic tool in HTPP, particularly in proximal phenotyping systems [37]. Genetic programming presented a good global classification success rate (75.2%) when compared with a random classifier (14 %). Thereby, the use of rapid fluorescence induction transients, handled by genetic programming, is considered promising for the rapid preliminary classification of *Vitis* genotypes.

## 4. Materials and Methods 

### 4.1. Plant Material

All measurements were made at the Portuguese *Vitis* germplasm bank from vineyards in the National Ampelographic Collection (NAC) at Estação Vitivinícola Nacional (Dois Portos), Portugal, in July 2017. The NAC was established in 1988 at INIAV-Dois Portos, 60 Km north of Lisbon and is the international reference for the *Vitis* genus in Portugal (reference—PRT 051). All the grapevine genotypes present (7 repetitions each) are maintained for the last 20 years and were originally collected in germplasm banks from France, Spain and Portugal. 

Three species of *Vitis* (*V. vinifera*, *V. rupestris* and *V. riparia*), one *Vitis* interspecific hybrid, Isabella (*Vitis labrusca* x *Vitis vinifera*) and four cultivars of *V. vinifera* (Pinot Noir, Cabernet Sauvignon, Riesling Weiss, Trincadeira) were sampled (Table 2). The same plants were used for both fluorescence measurement and genetic analysis. 

### 4.2. Genetic Analysis

Leaves were harvested from field grown plants, immediately frozen in liquid nitrogen and stored at −80 °C until use. DNA was extracted following the protocol described by Thomas et al. [39], with minor modifications. All the grapevine plants were genotyped with a set of nine Nuclear SSR Markers (Table 3) (VVS2, VVMD5, VVMD7, VVMD25, VVMD27, VVMD28, VVMD32, VRZAG62, VRZAG79) according to OIV for *Vitis* characterization [40]. These 9 SSR were divided in two multiplex mixes (Mplex1 SSRs: VVMD27, VVMD25, VVMD28 and VVMD32; Mplex2 SSRs: VVMD5, VVMD7 and VVS2) [41]. Each multiplex reaction was prepared according to the manufacturer’s instructions of Maxima Hot Start PCR Master Mix (2×).

PCR reactions were carried out with a final concentration of 200 μM of each dNTP (deoxynucleotide), 0.5 U of Taq DNA polymerase (Thermo Scientific, Waltham, MA. USA), 2 μL of 10× PCR buffer [(NH_4_)_2_SO_4_—Thermo Scientific, Waltham, MA, USA], 2.5 mM MgCl_2_ and 0.3 μM of each primer (0.125 μM for VVS2) and 10 ng DNA, to a final volume of 20 μL. Thermal cycling started with a denaturation step at 95 °C for 5 min followed by 35 cycles with a temperature profile of 95 °C for 20 s, 55 °C for 30 s and 72 °C for 30 s. For VVS2, 50 °C for 30 s and 72 °C for 5 min were used, respectively, for annealing temperature and final extension time. Capillary electrophoresis was carried out in the automatic sequencer CEQ 8000 Genetic Analysis System (Beckman Coulter, Brea, CA, USA). DNA size standard-400 (P/N 608109) was included as an internal sizing standard, and labeled products were analyzed and sized using the CEQ System (version 9) software, in order to determine their allelic sizes.

Molecular marker data analysis was done in order to confirm plant identity. The *Vitis vinifera* cultivars Pinot Noir and Cabernet Sauvignon were used to adjust alleles according *Vitis* International Catalogue of Varieties (VIVC, www.vivc.de, accessed March 2018) and to determine matching genotypes in VIVC. GENALEX v6.503 program package [47] was used to calculate the genetic distances among genotypes. MEGA 7 software (http://www.megasoftware.net) was used to construct a phylogenetic tree based on a matrix of genetic distances [48].

### 4.3. Fluorescence Measurements

In vivo chlorophyll a fluorescence was measured with a Handy Plant Efficiency Analyzer (PEA)—Chlorophyll Fluorimeter (Hansatech Instruments, Kings Lynn, UK). To minimize the interference of circadian rhythms, all measurements were performed in the middle of the day, between 12:00 and 16:00. Non-detached, sun-exposed, fully expanded healthy leaves were randomly selected and dark adapted for 10 min using light withholding leaf clips, always positioned to measure the upper side of the leaf. Samples were then exposed to a saturating light pulse with sufficient intensity to ensure closure of all PS II reaction centers (RC) (3500 µmolm^−2^s^−1^) for 1 s in order to obtain a chlorophyll a fluorescence transient rise (OJIP). Sixty leaves from three plants (twenty each) of each genotype were sampled once, providing 60 rapid fluorescence induction curves from each genotype. The curves comprise of 118 fluorescence vs. time data points, which are the attributes of each sample [27].

### 4.4. Machine Learning

K-nearest neighbors (KNN), decision trees (DT), neural networks (NN) and genetic programming (GP) were applied to rapid fluorescence induction curves recorded in the grapevine genotypes. This diverse set of methods represent four of the five “tribes” of machine learning recently identified by Domingos [49]. KNN does not produce an explicit model of the data but quickly reveals if the features have a spatial structure that will allow for the classification of unseen data based on the distance to the training data; DT is the logical first approach, as it represents the simplest method of providing a fully interpretable predictive model of the data; NN and GP are powerful methods that should only be used when a simpler method like DT does not provide good results.

For KNN, we used the Statistics and Machine Learning toolbox of MATLAB (R2018a). For DT and NN, we used the implementations included in the python library scikit-learn [50]. For GP, we used the multiclass M3GP classifier included in the GPLAB toolbox [51] for MATLAB. Regarding KNN, the number of neighbors k was optimized by the toolbox, and k = 5 was adopted as the number that resulted in better generalization, all the rest being the default parameters. Regarding DT, the library allows for the selection of criterion used to choose the attribute for each tree node, the maximum depth of the tree, and the minimum number of samples that an inner node can have (if all samples belong to the same class, then the node becomes a leaf node). We tested the entropy and Gini selection criteria; the maximum tree depth varied between 5 and 20, and the minimum number of samples in a node was 2, 5, 10 or 20. The best generalization results were determined to be the entropy criterion, maximum tree depth 19 and minimum number of samples 5. Regarding NN, the scikit-learn implementation of multilayer perceptron allows for the selection of the activation function used in neurons in inner layers, the number of inner layers, and the number of neurons in each layer. The number of neurons in a single inner layer was one of 20, 50 100, 200, 500, 1000, 2000, or 5000; the activation function was either the logistic sigmoid, hyperbolic tangent, or rectified linear. The best generalization was achieved with 5000 neurons on a single hidden layer, with the logistic activation function. The learning algorithm ran for 1,000,000 iterations at most or stopped if the success rate of the NN was higher than 99.99%. Other parameter settings used the default values. For the GP classifier, we used a population of 250 individuals and let it evolve for 100 generations, or until the accuracy reached 100% on the training set (which normally occurred around generation 50). All other settings were default ones. Unlike standard GP [52], which is not adequate for multiclass classification, the M3GP classifier [53,54] was specifically developed to solve problems involving multiple classes. It evolves hyper-features from original ones, transforming the original feature space into a new n-dimensional feature space, where n is also automatically found by the evolutionary process. Clusters are formed in this new space, one per class, and the predicted label of each observation is that of the nearest centroid, based on the Mahalanobis distance.

In all classifier methods, the complete Kautsky dataset was split into training dataset (90% of total samples) and test dataset (10% of total samples). The training dataset was used by the algorithm to obtain a classifier, and then we measured the success rate using the test dataset. We performed this random data split 30 times, and each time we trained and tested a new classifier, in order to arrive at a robust estimate of what level of accuracy is expected on unseen data.

## Figures and Tables

**Table 1:** Success rate and parameterization of the machine learning methods used.

Method	Success Rate	Main Parameters
K-nearest neighbors	58.5%	Number of neighbors: 5
Decision tree	51.6%	Split criterion: entropy Maximum tree depth: 19 Minimum number of samples in a node: 5
Neural network	71.8%	Number of neurons: 5000 Activation function: logistic
Genetic programming	75.3%	Number of individuals: 250 Number of generations: 100

**Table 2:** Grapevine genotypes used in this study. Grapevine species, variety and *V. vinifera* name, accession on the Portuguese National Ampelographic Collection, number on the *Vitis* International Variety Catalogue [38], leaf traits and country of origin are shown.

Genotype	Variety	Acession PRT051	VIVC	Photo (VIVC) *	Leaf Colour	Leaf Bright	Country of Origin
*Vitis rupestris* Scheele	Rupestris du Lot	13,821	10,389	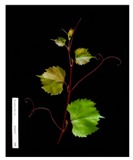	Light green	bright	France
*Vitis riparia* Michaux	Riparia Gloire de Montpellier	13,822	4824	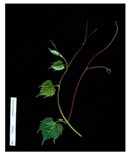	Dark green	dull	France
*Vitis* interspecific crossing	Isabella	13,619	5560	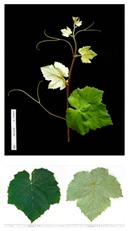	Dark green	dull	United States of America
*Vitis vinifera* Linné subsp. vinifera	Pinot Noir	10,918	9279	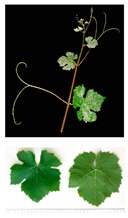	green	dull	France
*Vitis vinifera* Linné subsp. vinifera	Cabernet Sauvignon	10,714	1929	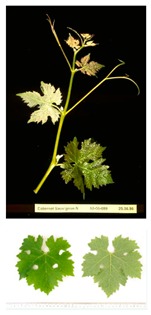	Light green	Slightly bright	France
*Vitis vinifera* Linné subsp. vinifera	Riesling Weiss	13,413	10,077	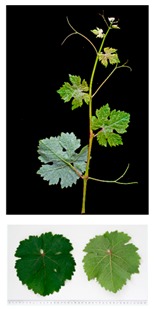	Dark green	Slightly bright	Germany
*Vitis vinifera* Linné subsp. vinifera	Trincadeira	11,402	15,685	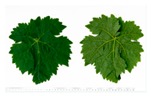	Dark green	Very bright	Portugal

* Photo credit: Julius Kühn-Institut, Institute for Grapevine Breeding Geilweilerhof, Germany—Vitis International Variety Catalogue—www.vivc.de—(September 2019).

**Table 3:** Name, linkage group, microsatellite sequences and references of the simple sequence repeats (SSRs) markers used in this study.

SSR Name	Linkage Group	Microsatellite Repeat Motif	Reference
VVS2	11	(GA)n	Thomas and Scott [42]
VVMD5	16	(CT)nAT(CT)nATAG(AT)n	Bowers and Meredith [43]
VVMD7	7	(CT)n	Bowers and Meredith [43]
VVMD25	11	(CT)n	Bowers et al. [44]
VVMD27	5	(CT)n	Bowers et al. [44]
VVMD28	3	(CT)n	Bowers et al. [44]
VVMD32	4	(CT)n	Bowers et al. [44]
VRZAG62	7	(GA)n	Sefc et al. [45]/Doligez et al. [46]
VRZAG79	5	(GA)n	Sefc et al. [45]/Doligez et al. [46]
